# Changbai Mountain Ginseng (*Panax ginseng* C.A. Mey) Extract Supplementation Improves Exercise Performance and Energy Utilization and Decreases Fatigue-Associated Parameters in Mice

**DOI:** 10.3390/molecules22020237

**Published:** 2017-02-05

**Authors:** Guo-Dong Ma, Chun-Hui Chiu, Yi-Ju Hsu, Chien-Wen Hou, Yi-Ming Chen, Chi-Chang Huang

**Affiliations:** 1 Sport Science College, Jilin Sport University, Changchun 130022, Jilin, China; magd2008@126.com; 2 Graduate Institute of Health Industry Technology, Research Center for Industry of Human Ecology and Research Center for Chinese Herbal Medicine, College of Human Ecology, Chang Gung University of Science and Technology, Taoyuan 33303, Taiwan; chchiu@mail.cgust.edu.tw; 3 Graduate Institute of Sports Science, National Taiwan Sport University, Taoyuan 33301, Taiwan; 1041302@ntsu.edu.tw; 4 Laboratory of Exercise Biochemistry, Department of Sports Sciences, University of Taipei, Taipei 11153, Taiwan; om65726@yahoo.com.tw

**Keywords:** ginsenoside, Ro, exercise performance, anti-fatigue, muscle mass

## Abstract

Changbai Mountain Ginseng (CMG, *Panax ginseng* C.A. Mey) is a traditional medicine commonly found in Northeast China and grows at elevations of 2000 m or higher in the Changbai Mountain Range. CMG, considered to be a “buried treasure medicine”, is priced higher than other types of ginseng. However, few studies have demonstrated the effects of CMG supplementation on exercise performance, physical fatigue, and the biochemical profile. The major compound of CMG extract was characterized by electrospray ionization tandem mass spectrometry (HPLC-ESI-MS/MS). Male ICR mice were divided into 3 groups, the vehicle, CMG-1X and CMG-5X groups (*n* = 8 per group), and respectively administered 0, 5, or 25 mg/kg/day of CMG extract orally for four weeks. HPLC-ESI-MS/MS results showed that the major compound in CMG extract is ginsenoside Ro. CMG extract significantly increased muscle weight and relative muscle weight (%). CMG extract supplementation dose-dependently increased grip strength (*p* < 0.0001) and endurance swimming time, decreased levels of serum lactate (*p* < 0.0001), ammonia (*p* < 0.0001), creatine kinase (CK, *p* = 0.0002), and blood urea nitrogen (*p* < 0.0001), and economized glucose levels (*p* < 0.0001) after acute exercise challenge. The glycogen in the gastrocnemius muscle was significantly increased with CMG extract treatment. Biochemical profile results showed that creatinine and triacylglycerol significantly decreased and total protein and glucose increased with CMG treatment. This is the first report that CMG extract supplementation increases muscle mass, improves exercise performance and energy utilization, and decreases fatigue-associated parameters in vivo. The major component of CMG extract is ginsenoside Ro, which could be a potential bioactive compound for use as an ergogenic aid ingredient by the food industry.

## 1. Introduction

Changbai Mountain ginseng (*Panax ginseng* C.A. Mey, CMG) is a wild ginseng that grows in the Changbai Mountain Range in Jilin Province, the highest mountains (2750 m) in Northeastern China. CMG is harvested from the local forests and has been used as a traditional Chinese medicine (TCM) for more than a century. Recently, the Chinese government has developed large wild ginseng growing bases, where traditional Chinese herbs are produced, to meet the Good Agricultural Practices (GAP) standards and encourage the development of the pharmaceutical industry in Northern China [1]. In general, the two major species of ginseng are Asian (*Panax ginseng*) and American (*Panax quinquefolius*) [2]. In previous reports, a common mixture of active ingredients was demonstrated to be ginsensosides [3,4,5]. The benefits of ginseng are attributed to bioactive compounds, such as ginsenosides, volatile oils, polyphenols, flavonoids, polysaccharides, and vitamins [6,7,8,9,10]. In previous reports on physiological functions, ginseng exhibited anti-cancer [11], immunomodulatory [12], and antidiabetic [13] effects, and cardiovascular improvement [14] in delivering ginseng ginsenosides. Ginseng roots vary from different regions, and differences in environmental factors could influence the genotypes [15]. Thus, CMG could be more bioactive than ginseng grown in other locations. The aim of the current study was to evaluate the anti-fatigue activities of ginseng polysaccharides, as well as the neutral, acidic portions and ginseng polysaccharides of CMG [16]. However, there is a lack of research to support the anti-fatigue effects of the major compounds of CMG, which could improve exercise performance. In this study, we identified the major compound of CMG extract and evaluated the ergogenic, anti-fatigue, and health promotion effects of CMG supplementation using our previously established in vivo platform [17,18].

## 2. Results

### 2.1. Characterized the Major Compound of CMG Extract by HPLC-ESI-MS/MS

**Figure 1:**
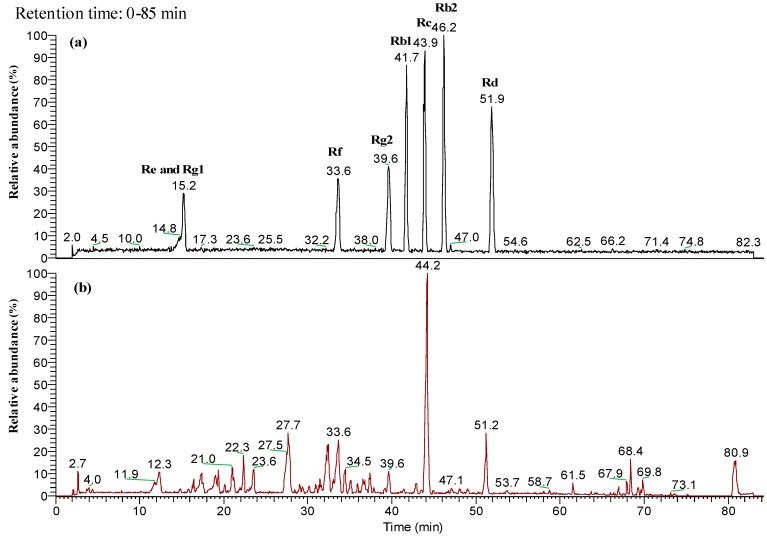
Base peak HPLC-ESI-MS chromatogram of ginsenosides in CMG extract. Ginseng ginsenoside mix standard (**a**, upper panel); CMG extract (**b**, bottom panel).

**Figure 2:**
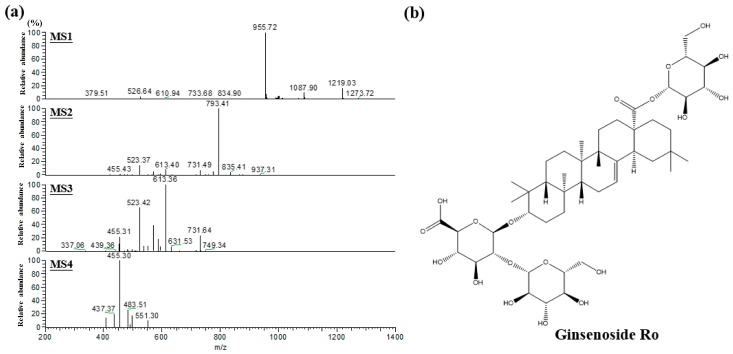
Characterized CMG extract by HPLC-ESI-MS/MSn. HPLC-ESI-MS/MSn chromatogram of major ginsenoside in CMG extract (**a**); and the chemical structure of the major compound in CMG extract (**b**).

CMG was systematically characterized by electrospray ionization tandem mass spectrometry (HPLC-ESI-MS/MSn). Figure 1a shows the ginsenoside standard chromatogram (Rc, Rb2, Rd, Rg2, Rb1, Rf, Re, Rg1); no ginsenoside Rc, Rb2, Rd, Rg2, Rb1, Rf, Re, Rg1 were found in the CMG extract (Figure 1b). The major compound was found at a 44.2 min retention time. The major parent ion was detected and processed for multiple fragment analysis. As shown in Figure 2a, the MS1 parent ions were identified as 955 *m*/*z* [M − H]^−^, the MS2 secondary ions were 793 *m*/*z* [M − H − glc]^−^, the MS3 ions were 613 *m*/*z* [M − H − 2glc − H_2_O]^−^, and the MS4 ions were 455 *m*/*z* [M − H − 2glc − glcA]^−^, which were oleanolic acid aglycone. Above all, the major compound in CMG was ginsenoside Ro, which is consistent with a previous study [19]. The structure of that compound is shown in Figure 2b.

### 2.2. Effects of CMG on Forelimb Grip Strength

**Figure 3:**
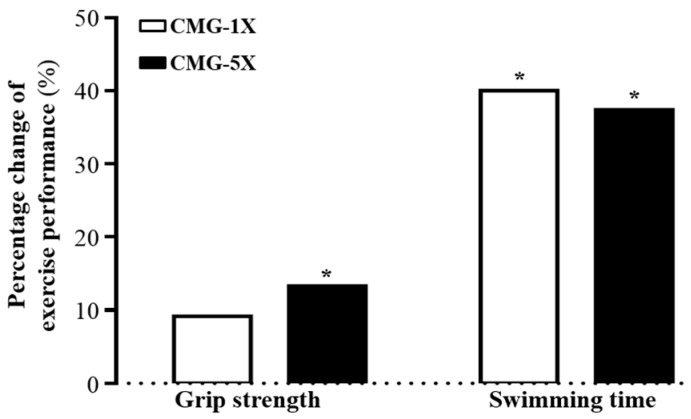
Percentage change with vehicle treatment of CMG (Changbai Mountain Ginseng) supplementation for four weeks on forelimb grip strength and swimming time. Mice were pretreated with vehicle, CMG-1X, or CMG-5X for four weeks before forelimb grip strength was tested. Asterisk (*), significant difference (*p* < 0.05) from vehicle group.

After four weeks of CMG supplementation, the forelimb grip strength (Table 1, Figure 3) was 1.27-fold higher in the CMG-5X mice than in the vehicle treatment mice (*p* < 0.0001). In the trend analysis, absolute forelimb grip strength dose-dependently increased with increasing CMG dose (*p* < 0.0001).

### 2.3. Effect of CMG on Exercise Performance in a Weight-Loaded Swimming Test

Energy metabolism during muscular activity determines the level of physiological fatigue, and exercise endurance is an important index for evaluating anti-fatigue treatment. Endurance swimming times were 2.5 ± 0.4, 4.1 ± 1.4, and 4.0 ± 1.1 min with the vehicle, CMG-1X, and CMG-5X treatments, respectively (Table 1, Figure 3). The endurance swimming times were respectively 1.67- and 1.60-fold longer in the CMG-1X and CMG-5X groups than in the vehicle treatment group (*p* = 0.0055 and *p* = 0.0196, respectively). 

### 2.4. Effect of CMG Supplementation on Serum Lactate, Ammonia, Glucose, CK, and BUN Levels after Acute Exercise Challenge

**Figure 4:**
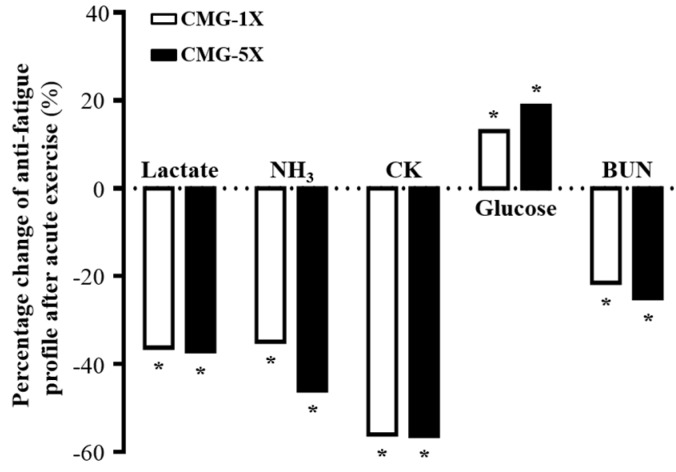
Percentage change with vehicle treatment of CMG supplementation on serum levels of lactate, ammonia, creatine kinase (CK) , glucose, and blood urea nitrogen (BUN) after acute exercise challenge. Asterisk (*), significant difference (*p* < 0.05) from vehicle group.

In the present study, we determined anti-fatigue related serum levels after 15 min swimming (Table 2, Figure 4). Lactate levels were significantly lower in the CMG-1X and CMG-5X supplementation groups (36.2%, *p* < 0.0001 and 37.0%, *p* < 0.005, respectively) than in the vehicle treatment group, indicating that CMG supplementation was a factor in the removal and utilization of blood lactate after exercise. Serum ammonia levels were respectively 34.9% (*p* = 0.0123) and 45.9% (*p* = 0.0017) lower in the CMG-1X and CMG-5X groups than in the vehicle treatment group. The trend analysis revealed that lactate and serum ammonia level dose-dependently decreased with increasing CMG dose (*p* < 0.0001). Thus, continuous supplementation with CMG for four weeks decreased lactate and ammonia levels during exercise. Serum CK level is an important clinical biomarker of muscle damage. Therefore, reduced serum CK levels could represent increased recovery capacity from exercise-induced muscle damage. CK levels were respectively 55.9% and 56.3% (*p* < 0.0001) lower in the CMG-1X and CMG-5X groups than in the vehicle group, indicating that CMG supplementation may ameliorate skeletal muscle injury induced by acute exercise challenge.

Blood glucose level is an important index for performance maintenance during exercise. The glucose level was 1.4-fold higher (*p* = 0.0001) in the CMG-5X group than in the vehicle group (Figure 4). The trend analysis showed that serum glucose levels dose-dependently increased with CMG supplementation dose (*p* < 0.0001). BUN is an important biochemical parameter related to fatigue. The BUN level is a measure of the amount of nitrogen in blood from the waste product of urea. Urea serves an important role in the metabolism of nitrogen-containing compounds. Serum BUN levels were respectively 21.4% and 25.0% (*p* < 0.0001) lower in the CMG-1X and CMG-5X groups than in the vehicle group. Trend analysis showed that absolute BUN level decreased dose-dependently with increases in CMG dose (*p* < 0.0001). Therefore, CMG may be considered as an ergogenic supplement that facilitates the removal of metabolic waste during exercise.

### 2.5. Effect of CMG Supplementation for 4 Weeks on Hepatic and Muscle Glycogen Levels

**Figure 5:**
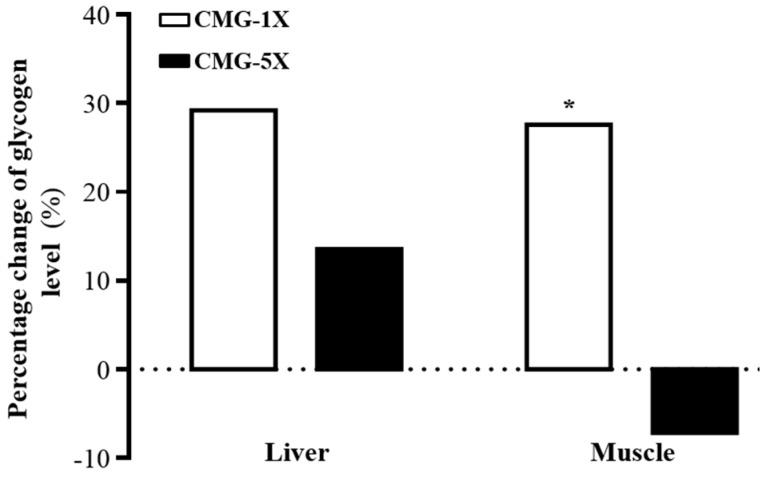
Percentage change with vehicle treatment of CMG supplementation on glycogen levels in liver and muscle. Asterisk (*), significant difference (*p* < 0.05) from vehicle group.

Glycogen storage directly affects exercise ability, so we measured the glycogen contents of liver and muscle tissues (Table 3). The liver glycogen content suggested no significant differences with all treatments (Figure 5); however, the liver glycogen was slightly higher in the supplementation groups than in the vehicle group. On the other hand, the muscle glycogen content was 1.38-fold (*p* = 0.0073) higher in the CMG-1X group than in the vehicle group. Thus, CMG-1X slightly increased liver and muscle glycogen storage.

### 2.6. General Characteristics of Mice with CMG Supplementation for Four Weeks

Initial BW did not differ among the vehicle, CMG-1X, and CMG-5X groups (Table 4). After four-week supplementation with CMG, the final BW was not different in each group. Daily intake of diet and water was not different in the vehicle and CMG supplementation groups, either. Thus, four-week supplementation with CMG affected neither BW nor water and diet intake. We also measured the effect of CMG on muscle mass and relative muscle weight (different tissue weights adjusted for individual BW %). The muscle weights (gastrocnemius and soleus muscles) were, respectively, 1.08- (*p* = 0.0021) and 1.09-fold (*p* = 0.0005) higher in the CMG-1X and CMG-5X groups than in the vehicle group. Trend analysis showed that muscle weight dose-dependently increased with CMG supplementation (*p <* 0.0001). The relative muscle weights (%) were, respectively, 1.17- (*p* = 0.021) and 1.23-fold (*p* = 0.0032) greater in the CMG-1X and CMG-5X groups than in the vehicle group. Trend analysis also showed significant dose-dependent increases in muscle weight (*p* < 0.0001) and relative muscle weight (*p* = 0.0004) with CMG supplementation. In addition, the BAT weights were, respectively, 1.18- (*p* = 0.006) and 1.24-fold (*p* = 0.0006) higher in the CMG-1X and CMG-5X groups than in the vehicle group. Trend analysis revealed that BAT weight and relative BAT weight (both *p* < 0.0001) had significant dose-dependent effects with CMG supplementation. Thus, supplementation with CMG for four weeks could be beneficial to muscle mass and BAT weight. According to this data, we found no gross abnormalities attributed to CMG when weighing organs.

### 2.7. Effect of CMG Supplementation on Biochemical Variables at the End of the Experiment

We observed beneficial effects of CMG on grip strength, exhaustive exercise challenge, and body composition with four-week CMG supplementation. We further investigated whether four-week CMG treatment affected other biochemical markers in healthy mice (Table 5).

**Figure 6:**
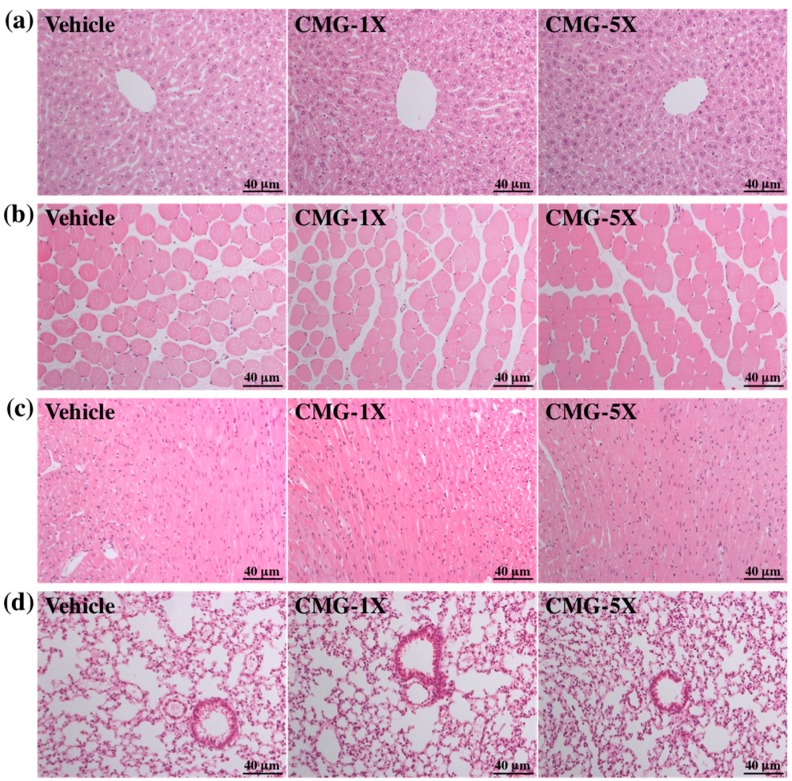

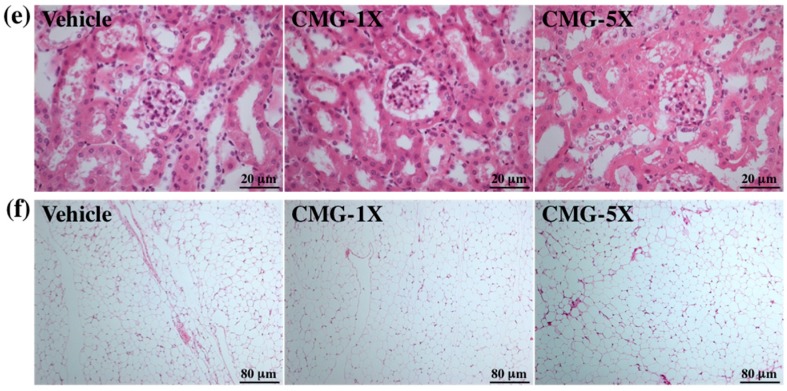
Effect of CMG supplementation on morphology of liver (**a**); skeletal muscle (**b**); heart (**c**); lungs (**d**); kidney (**e**); and epididymal fat pad (**f**). Specimens were photographed by light microscopy. (H and E stain, magnification: (**a**–**d**), 200×, Scale bar, 40 µm; (**e**), 400×, Scale bar, 20 µm; (**f**), 100×, Scale bar, 80 µm).

Levels of biochemical indices, including aspartate aminotransferase (AST), alanine aminotransferase (ALT), lactate dehydrogenase (LDH), blood urea nitrogen (BUN), creatinine, uric acid (UA), and total cholesterol (TC), were the same among groups (*p* > 0.05, Table 2). Serum CK levels were 26.63% (*p =* 0.0455) lower with CMG-5X than with vehicle treatment. Serum TP and glucose levels were respectively 1.06- (*p =* 0.0268) and 1.14-fold (*p =* 0.0145) higher with CMG-5X than with vehicle treatment. Serum TG levels were significantly lower with CMG-1X (*p* = 0.0025) and CMG-5X (*p* = 0.0022) than with vehicle treatment. Trend analysis showed that serum CK (*p* = 0.0045) and TG (*p* < 0.0001) levels dose-dependently decreased with CMG extract supplementation; serum TP (*p* = 0.0017) and glucose (*p* = 0.0030) dose-dependently increased with CMG extract supplementation. In addition, serum albumin slightly increased with CMG-5X treatment, while the albumin levels were still within the normal range. Therefore, long-term daily supplementation with CMG extract may have the potential to protect tissue and enhance nutrient absorption, with increases in serum TP and glucose level. We also examined the tissue morphology. CMG supplementation for four weeks had no adverse effects on major organs such as the liver, skeletal muscle, heart, kidney, lung, and epididymal fat pad (EFP). Therefore, the dose of CMG extract supplementation used in this study was safe (Figure 6).

## 3. Discussion

The beneficial effects of ginsenoside species have been well demonstrated in many studies. However, the function of ginsenoside Ro, an oleanane-type saponin, has not been sufficiently investigated. The place of origin and weather could influence ginseng trace elements and secondary metabolites, which could explain why CMG differs from other types of ginseng and has a high content of ginsenoside Ro. In general, programmed exercise training is required to increase grip strength [20]; however, we found that ginsenoside Ro supplementation benefited grip strength even though the test animals underwent no training intervention. Thus, long-term ginsenoside Ro supplementation could benefit the muscle explosive force under non-training conditions. However, few studies have investigated the use of ginseng supplementation to improve muscle strength. According to a previous study, dammarane steroid, a ginseng steroid that is present in many ginseng species, has anti-inflammatory effects on skeletal muscle following a bout of muscle-damaging exercise [21]. Thus, ginsenoside Ro may have an anti-inflammation effect and enhance skeletal muscle performance. Ginseng has a wide range of benefits that could promote physical performance and recovery capacity from interval exercise [22]. We suggest that ginsenoside Ro may improve endurance performance in the absence of training. Further investigation is required to clarify which types of saponins could benefit exercise training for endurance and explosive force performance. In a previous report, 20(R)-ginsenoside Rg3 was demonstrated to increase weight-loaded swimming time [23]. Moreover, ginsenoside Ro may act as a potential ergogenic aid for exercise supplementation.

Exercise-induced muscle fatigue can be evaluated with biochemical indicators, including lactate, ammonia, glucose, CK, and BUN levels [24,25]. The clearance of lactate is thought to reduce peripheral neuromuscular fatigue and have positive effects on muscle function [26]. After acute exercise, relaxation is significantly affected by the blood lactate clearance rate. Approximately 75% of the total amount of lactate produced is used for oxidative production of energy in the exercising body, and lactate could be utilized for the de novo synthesis of glucose in the liver [27]. During exercise, muscle utilizes glucose from glycogen by intramuscular glycogenolysis and by increased glucose uptake. Regardless, aerobic and resistance exercises increase glucose transporter type 4 (GLUT4) abundance and translocation, thereby increasing serum glucose uptake by a pathway that is not dependent on insulin [28,29]. Ammonia, an important metabolite during energy metabolism for exercise, is generated by different sources. Accumulation of ammonia in the blood and brain during exercise can negatively affect the central nervous system and cause fatigue. The present data suggest that continuous supplementation with CMG extract for four weeks could decrease lactate, ammonia, and BUN accumulation, economize serum glucose levels and, thus, CMG extract could have anti-fatigue activity. The effect on CK serum response appears to be related to the magnitude of eccentric contractions involved in the activity and the subsequent extent of muscle disruption [30]. High-intensity exercise challenge can physically or chemically cause tissue damage and muscular cell necrosis [31]. Thus, ginsenoside Ro could reduce muscle damage after exercise.

Concerning glycogen, in our present study, ginsenoside Ro may have influenced hepatic and muscle glycogen metabolism. Previously, it has been reported that ginsenoside suppresses hepatic glucose production through inhibiting the small heterodimer partner (SHP) gene expression [32] or increasing hepatic glycogen storage [33]. Ginseng, or ginsenosides associated with acetyl CoA carboxylase kinase (AMPK) activation, switches off anabolic pathways, including glycolysis, lipolysis, glycogen synthesis, and protein synthesis in the liver [34]. There are at least three mechanisms to regulate the glucose uptake of skeletal muscle. To begin with, glucose delivery to the skeletal muscle cells; then, glucose transport through the cell surface membrane; and, finally, flux through intracellular metabolism [35]. During short-term exercise, the muscle glycogen is the preferred carbohydrate fuel for both aerobic and anaerobic metabolism. When exercise is prolonged, glycogen stores in muscle and liver are depleted during exercise, and blood glucose utilization becomes the main carbohydrate fuel during exercise [36]. Therefore, we suggested that CMG-1X supplementation was the proper dose for recommendation as an ergogenic aid. Our present data also showed that CMG-5X could increase the serum glucose levels by decreasing glycogen storage. We suggested that the ginsenosides may increase glycolysis [37]. During exercise, both serum glucose and muscle glycogen are important fuels. The increase of muscle glucose uptake during exercise depends upon the delivery of glucose (capillary perfusion and plasma glucose concentration) and the permeability of the muscle membrane to glucose [35]. In addition to muscle glycogen content, serum glucose level is the other energy utilization index. Therefore, CMG could modulate the muscle glycogen storage and ginsenoside Ro could be as an ergogenic supplement. It had been reported that ginseng saponin decreases plasma triglyceride levels and inhibits atheroma formation in animals with hypercholesterolemia [38]. The reason why enhanced nutrition absorption with TP was increased by CMG extract supplementation could be that the ginsenosides were biotransformed by intestinal bacteria, which further improved intestinal absorption and bioactivity and diminished the toxicity of the metabolite [39,40].

However, there were still some imitations of this study. The major limitation of the study is that we cannot extrapolate our results to the human because only animal blood of tissue samples were included in this study. Another limitation of the study is the lack of information on the major compound of ginsenoside profiling data from fresh or dried material or during growth of Changbai Mountain Ginseng in different seasons.

## 4. Materials and Methods

### 4.1. Preparation of Changbai Mountain Ginseng (CMG) Extract

CMG specimens were cut into small pieces and soaked in ethanol at ambient temperature for seven days. The extracts were decanted and filtered through Whatman No. 2 filter paper (Sigma, St. Louis, MO, USA), and the filtrates were concentrated in a rotary evaporator before being lyophilized. The yield of CMG was 0.4% (1.96 g/470.5 g × 100).

### 4.2. Liquid Chromatographic-Mass Spectrometry (LC-MS) Analysis of CMG Extract

One hundred microliters of CMG extract powder (670 mg/mL) were dissolved in milli-Q water. Before being loaded into SPE (polymeric reversed-phase solid-phase-extraction cartridges; 200 mg/3 mL; StrataX, Phenomenex, Denver, CO, USA), the CMG extract liquid was added into 600 μL methanol and then eluted out with 10%–100% of acetonitrile. The system included an HPLC equipped with a Thermo Finnigan model LXQ linear ion trap mass spectrometer (San Jose, CA, USA) operated in negative ion electrospray mode. A YMC Hydrosphere C18 analytical column (2.0 × 150 mm, 5 μm, YMC, Kyoto, Japan) was maintained at room temperature, and a flow-rate of 0.2 mL/min was used. The mobile phase consisted of water with 0.1% formic acid (solvent A) and acetonitrile with 0.1% formic acid (solvent B). Gradients were programmed as follows: 21% B for 0–5 min, 21%–34% B for 5–40 min, holding at 34% B for 40–60 min, 34%–38% B for 60–70 min, 38%–100% B for 70–80 min, and holding at 100% B for 80–85 min. A ginseng ginsenosides standard kit (Sigma, G-015, St. Louis, MO, USA) was prepared for injection volumes of 10 µL.

### 4.3. Animals and Experiment Design

Male ICR mice (nine weeks old) grown under specific pathogen-free conditions were purchased from BioLASCO (Yi-Lan, Taiwan). All mice were provided a standard laboratory diet (No. 5001; PMI Nutrition International, Brentwood, MO, USA) and distilled water ad libitum, and they were housed with a 12-h light/12-h dark cycle at room temperature (22 ± 1 °C) and 50%–60% humidity. The Institutional Animal Care and Use Committee (IACUC) of National Taiwan Sport University (NTSU) inspected all animal experiments, and this study conformed to the guidelines of protocol IACUC-105,020 approved by the IACUC ethics committee. In this study, the dose of CMG for humans was 24.4 mg per day (CMG extract). The mice dose (5 mg/kg) we used was converted from a human-equivalent dose (HED) based on body surface area by the following formula from the US Food and Drug Administration: assuming a human weight of 60 kg, the HED for 24.4 (mg)/60 (kg) = 0.406 × 12.3 = 5 mg/kg; the conversion coefficient 12.3 was used to account for differences in body surface area between mice and humans as recently described [41]. In total, 24 mice were randomly assigned to three groups (eight mice/group) for daily oral CMG treatment for 4 weeks. The groups and treatment courses were as follows: vehicle; 5 mg/kg (CMG-1X); and 25 mg/kg (CMG-5X). The vehicle group received the same volume of solution equivalent to individual body weight (BW). Mice were randomly housed in groups of four per cage.

### 4.4. Forelimb Grip Strength Test

A low-force testing system (Model-RX-5, Aikoh Engineering, Nagoya, Japan) was used to measure the forelimb grip strength of treated mice as previously described [42]. Forelimb grip strength was tested one hour after administration of the indicated CMG supplementation for four weeks. The forelimb grip strengths of all mice were measured on the same day.

### 4.5. Swimming Exercise Performance Test

The swimming endurance test was conducted after the forearm grip strength test for all mice. For the swim-to-exhaustion test, loads corresponding to 5% of the mouse BW were attached to the tail to evaluate endurance time [43]. The swimming endurance time of each mouse was recorded from the beginning to exhaustion, determined by observing loss of coordinated movements and failure to return to the surface within 7 s. The test of swimming endurance time was performed one hour after administration of the indicated CMG supplementation for 28 days. The swimming exercise performance tests of all mice were performed on the same day as the forelimb grip strength measurement.

### 4.6. Determination of Fatigue-Associated Biochemical Variables

The effect of CMG supplementation on fatigue-associated biochemical indices was evaluated after exercise as previously described [43]. The 15-min swimming test was performed one day after the forelimb grip strength and the swimming exercise performance test. After CMG supplementation for one hour, all mice underwent a 15-min swimming test without weight loading. Immediately after the 15-min test, blood samples were immediately collected from the submandibular duct of mice and centrifuged at 1500× *g* and 4 °C for 10 min for serum preparation. Serum lactate, ammonia, glucose, creatine kinase (CK), and blood urea nitrogen (BUN) levels were determined with an autoanalyzer (Hitachi 7060, Hitachi, Tokyo, Japan).

### 4.7. Clinical Biochemical Profiles

Two days after the 15-min swimming test, all mice were sacrificed with 95% CO_2_ asphyxiation, and their blood was immediately collected. Blood collected by cardiac puncture was centrifuged at 1500× *g* for 10 min at 4 °C. Serum was separated by centrifugation and the levels of clinical biochemical variables (CK, albumin, total protein (TP), BUN, creatinine, uric acid (UA), total cholesterol (TC), triacylglycerols (TG), and glucose) were measured with an autoanalyzer (Hitachi 7060, Hitachi, Tokyo, Japan).

### 4.8. Histology of Tissues

Liver, skeletal muscle, heart, lung, kidney, and epididymal fat pad (EFP) tissues were carefully removed, minced, and fixed in 10% formalin. Samples were embedded in paraffin and cut into 4-μm thick slices for morphological and pathological evaluations. Tissues were stained with hematoxylin and eosin (H and E) and examined under a light microscope equipped with a CCD camera (BX-51, Olympus, Tokyo) by a veterinary pathologist.

### 4.9. Tissue Glycogen Determination and Visceral Organ Weight

The stored form of glucose is glycogen, which mostly exists in liver and muscle tissue. Liver and muscle tissues were excised after the mice were sacrificed and weighed for glycogen content analysis as described previously [44]. The weights of the liver, skeletal muscle, heart, lung, kidney, epididymal fat pad (EFP), and brown adipose tissue (BAT) related to visceral organs were recorded.

### 4.10. Statistical Analysis

All data are expressed as mean ± SD, *n* = 8 mice per group. Statistical differences among groups were analyzed with one-way analysis of variance (ANOVA and the Cochran-Armitage test for dose-effect trend analysis with SAS 9.0 (SAS Inst., Cary, NC, USA). *p* < 0.05 was considered statistically significant. Differences between groups were analyzed by one-way analysis of variance (ANOVA) using Duncan’s post-hoc test, and *p* values < 0.05 were considered significant.

## 5. Conclusions

**Figure A1:**
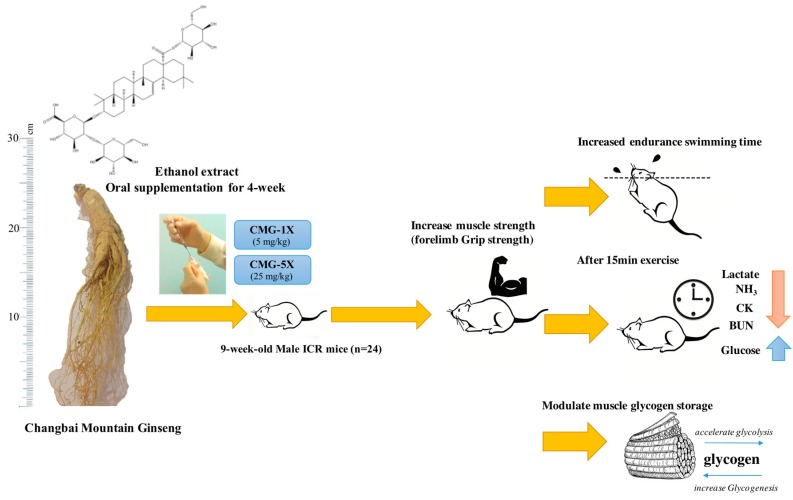
The Bioactivities of Changbai Mountain Ginseng (CMG) Extract on Anti-Fatigue In Vivo.

We demonstrated that the major compound of CMG is ginsenoside Ro. CMG supplementation for four weeks significantly improved forelimb grip strength and swimming time to exhaustion in test animals (Figure A1). CMG has anti-fatigue activity; it decreased plasma lactate, ammonia, and CK and increased serum glucose levels, thereby enhancing exercise performance in mice. Exercise-induced fatigue-related parameters including lactate, ammonia, CK, and glucose levels were dose-dependently modulated by CMG supplementation. CMG also has positive effects on the lipid profile, tissue protection, and nutritional status in vivo. In this study, we found that CMG supplementation could increase exercise performance, and increase muscle mass. These findings suggest potential biological mechanisms by which ginsenoside Ro may increase energy utilization via glycolysis and thereby increase serum glucose levels or decrease physical fatigue during exercise. Ginsenoside Ro may be associated with AMPK activation, which regulates metabolic processes, including glycolysis or glycogen synthesis. Previous studies have been demonstrated that ginsenoside has an anti-fatigue effect which related to improve energy metabolism and decrease the oxidative stress of skeletal muscle [45,46]. The underlying mechanism might be associated with an increase in the content of adenosine triphosphate (ATP) and an enhancement in the activity of energy metabolic enzymes, but the feasibility of other mechanisms still warrants further study. To our best knowledge, this is the first study to investigated that the ginsenoside Ro-rich CMG exhibits anti-fatigue function. Although we have evidence that revealst CMG could improve exercise performance, there is a lack of studies of ginsenoside-Ro in humans. Therefore, these results should be conducted to verify the biological effectiveness in human studies.

## Figures and Tables

**Table 1:** Effect of CMG (Changbai Mountain Ginseng) supplementation for four weeks on exercise performance.

Exercise Performance	Vehicle	CMG-1X	CMG-5X	Trend Analysis
Forelimb grip strength (g)	114 ± 13 ^a^	125 ± 10 ^a^	145 ± 14 ^b^	<0.0001 (↑)
Weight-loaded Swimming time (min)	2.5 ± 0.4 ^a^	4.1 ± 1.4 ^b^	4.0 ± 1.1 ^b^	0.0017 (↑)

All mice were sacrificed at the end of the experiment and examined for serum levels of clinical biochemistry. Values are mean ± SD for *n* = 8 mice per group. Values in the same line with different superscripts letters (a and b) differ significantly, *p* < 0.05 by one-way ANOVA. The arrows up mean dose-dependently increase by CMG supplementation.

**Table 2:** Effect of CMG supplementation on serum levels after acute exercise challenge.

Serum Levels after 15 min Swimming	Vehicle	CMG-1X	CMG-5X	Trend Analysis
Lactate(mmol/L)	7.3 ± 0.7 ^a^	4.7 ± 0.8 ^b^	4.6 ± 0.4 ^b^	<0.0001 (↓)
NH_3_ (μmol/L)	223 ± 95 ^a^	145 ± 15 ^b^	121 ± 20 ^b^	<0.0001 (↓)
CK (U/L)	907 ± 328 ^a^	400 ± 75 ^b^	397 ± 37 ^b^	0.0002 (↓)
Glucose (mg/dL)	125 ± 22 ^a^	143 ± 19 ^b^	177 ± 25 ^b^	<0.0001 (↑)
BUN (mg/dL)	34.6 ± 4.5 ^a^	27.2 ± 1.6 ^b^	25.9 ± 2.2 ^b^	<0.0001 (↓)

All mice were sacrificed at the end of experiment and examined for serum levels of clinical biochemistry. Values are mean ± SD for *n* = 8 mice per group. Values in the same line with different superscripts letters (a and b) differ significantly, *p* < 0.05 by one-way ANOVA. CK, creatine kinase; BUN, blood urea nitrogen. The arrows up (down) mean dose-dependently increase (decrease) by CMG supplementation.

**Table 3:** Effects of CMG supplementation on glycogen levels in liver and muscle.

Glycogen Contents	Vehicle	CMG-1X	CMG-5X	Trend Analysis
Liver (µg/g)	8.0 ± 4.0	11.3 ± 6.1	9.2 ± 2.0	0.3564
Muscle (µg/g)	0.16 ± 003 ^a^	0.23 ± 0.06 ^b^	0.15 ± 0.03 ^a^	0.4625

All mice were sacrificed at the end of experiment and examined for serum levels of clinical biochemistry. Values are mean ± SD for *n* = 8 mice per group. Values in the same line with different superscripts letters (a, b) differ significantly, *p* < 0.05 by one-way ANOVA.

**Table 4:** General characteristics of mice with Changbai Mountain Ginseng (CMG) supplementation.

Characteristic	Vehicle	CMG-1X	CMG-5X	Trend Analysis
Initial BW (g)	37.2.6 ± 1.0	37.3 ± 1.3	37.8 ± 1.7	0.2231
Final BW (g)	38.5 ± 1.5	38.9 ± 0.9	38.6 ± 1.3	0.7732
Food intake (g/day)	7.3 ± 0.6	7.2 ± 0.7	7.2 ± 0.3	0.8104
Water intake (mL/day)	8.5 ± 0.6	8.6 ± 0.5	8.3 ± 0.3	0.1808
Liver (g)	2.09 ± 0.16	2.10 ± 0.10	2.08 ± 0.14	08814
Kidney (g)	0.60 ± 0.05	0.60 ± 0.03	0.61 ± 0.05	0.7797
Heart (g)	0.26 ± 0.04	0.27 ± 0.03	0.27 ± 0.04	0.4513
Lung (g)	0.22 ± 0.02	0.22 ± 0.01	0.22 ± 0.02	0.9438
Muscle (g)	0.37 ± 0.02 ^a^	0.39 ± 0.01 ^b^	0.40 ± 0.01 ^b^	<0.0001 (↑)
EFP (g)	0.33 ± 0.15	0.31 ± 0.01	0.29 ± 0.01	0.8270
BAT (g)	0.076 ± 0.011 ^a^	0.090 ± 0.006 ^b^	0.095 ± 0.009 ^b^	<0.0001 (↑)
Relative liver weight (%)	5.43 ± 0.32	5.40 ± 0.30	5.38 ± 0.22	0.8766
Relative kidney weight (%)	1.56 ± 0.11	1.55 ± 0.08	1.57 ± 0.13	0.9078
Relative Heart weight (%)	0.67 ± 0.10	0.70 ± 0.08	0.69 ± 0.10	0.4836
Relative Lung weight (%)	0.58 ± 0.05	0.56 ± 0.03	0.58 ± 0.05	1.0000
Relative Muscle weight (%)	0.95 ± 0.05 ^a^	1.01 ± 0.03 ^b^	1.03 ± 0.05 ^b^	0.0004 (↑)
Relative EFP weight (%)	0.85 ± 0.37	0.79 ± 0.18	0.76 ± 0.20	0.8081
Relative BAT weight (%)	0.20 ± 0.03 ^a^	0.23 ± 0.02 ^b^	0.25 ± 0.03 ^b^	<0.0001 (↑)

Data are mean ± SD, *n* = 8 mice/group. Values in the same line with different superscripts letters (a, b) differ significantly, *p* < 0.05 by one-way ANOVA. Food efficiency ratio: body weight (BW) gain (g/day)/food intake (g/day). Muscle mass includes both gastrocnemius and soleus muscles in the lower legs. BAT: brown adipose tissue; EFP: epididymal fat pad. Mice were pretreated with vehicle, CMG-1X, or CMG-5X for 4 weeks. The arrows up mean dose-dependently increase by CMG supplementation.

**Table 5:** Biochemical analysis with CMG supplementation at the end of the experiment.

Variables	Vehicle	CMG-1X	CMG-5X	Trend Analysis
AST (U/L)	102 ± 14	102 ± 11	101 ± 11	0.5837
ALT (U/L)	61 ± 16	59 ± 19	60 ± 22	0.7688
LDH (U/L)	534 ± 60	528 ± 82	537 ± 76	0.9100
CK (U/L)	560 ± 140 ^b^	507 ± 148 ^ab^	413 ± 124 ^a^	0.0045 (↓)
TP (g/dL)	4.9 ± 0.2 ^a^	5.0 ± 0.4 ^ab^	5.2 ± 0.2 ^b^	0.0017 (↑)
Albumin (g/dL)	3.0 ± 0.1 ^a^	3.0 ± 0.1 ^a^	3.2 ± 0.1 ^b^	<0.0001 (↑)
BUN (mg/dL)	23.6 ± 1.8	23.9 ± 1.6	22.5 ± 1.7	0.4280
Creatinine (mg/dL)	0.30 ± 0.03	0.31 ± 0.04	0.30 ± 0.02	0.8319
UA (mg/dL)	1.9 ± 0.6	1.7 ± 0.3	1.7 ± 0.3	0.5904
TC (mg/dL)	137 ± 13	133 ± 8	131 ± 8	0.3105
TG (mg/dL)	153 ± 15 ^b^	124 ± 22 ^a^	124 ± 11 ^a^	<0.0001 (↓)
Glucose (mg/dL)	148 ± 18 ^a^	155 ± 16 ^ab^	168 ± 10 ^b^	0.0030 (↑)

Data are mean ± SD, *n* = 8 mice/group. Values in the same line with different superscripts letters (a, b) differ significantly, *p* < 0.05 by one-way ANOVA. AST, aspartate aminotransferase; ALT, alanine aminotransferase; LDH, lactate dehydrogenase; CK, creatine kinase; TP, total protein; BUN, blood urea nitrogen; UA, uric acid; TC, total cholesterol; TG, triacylglycerols. The arrows up (down) mean dose-dependently increase (decrease) by CMG supplementation.
